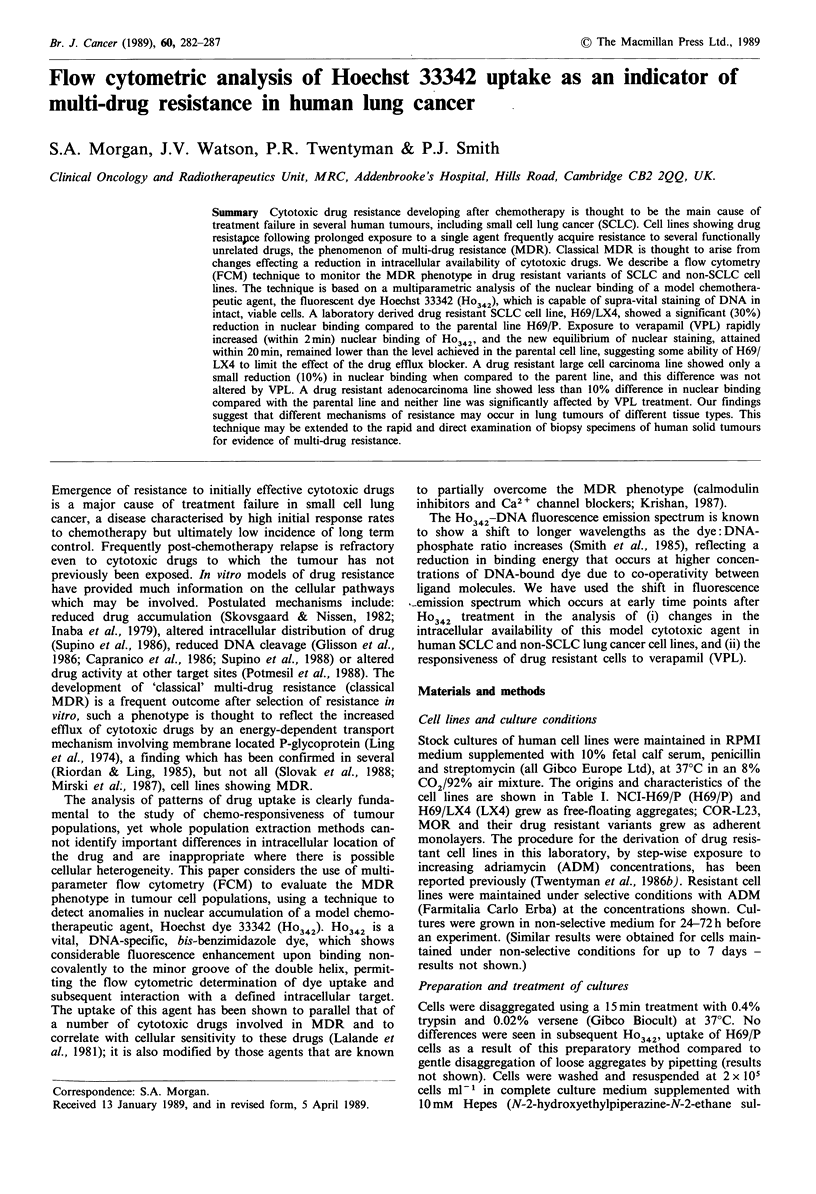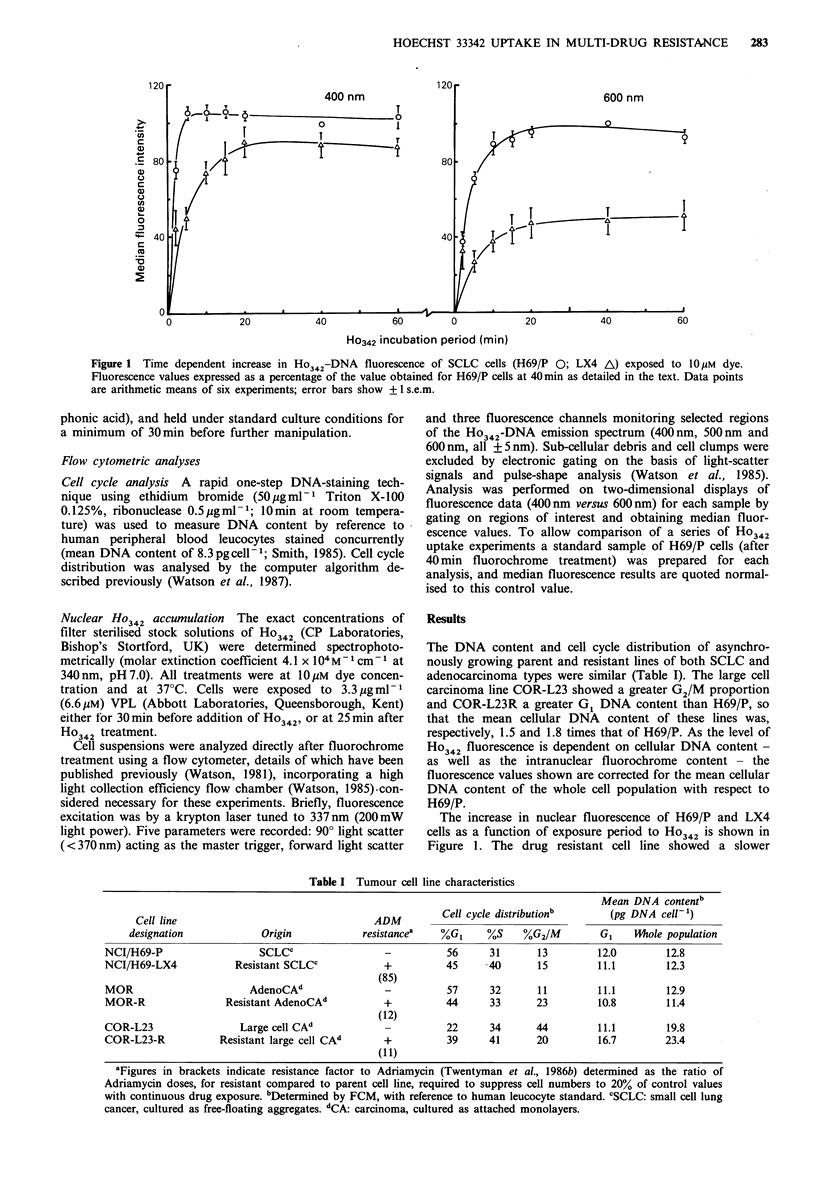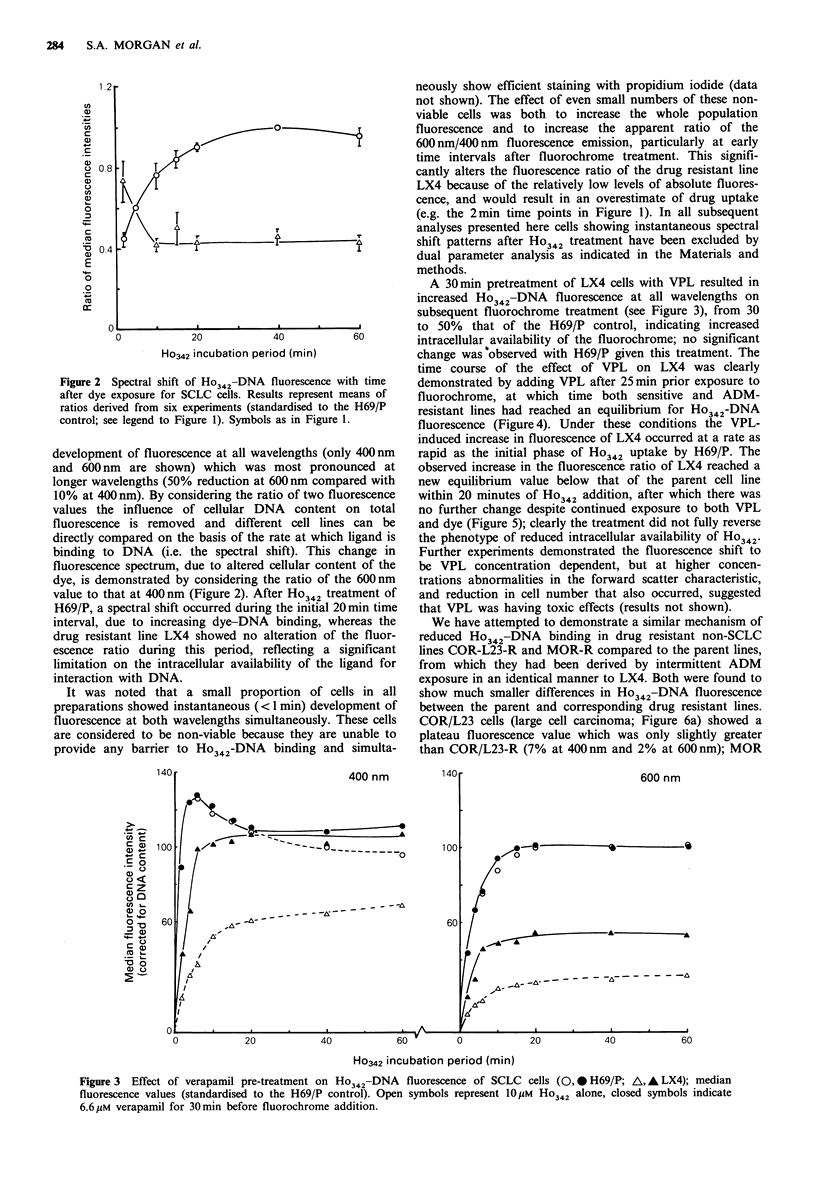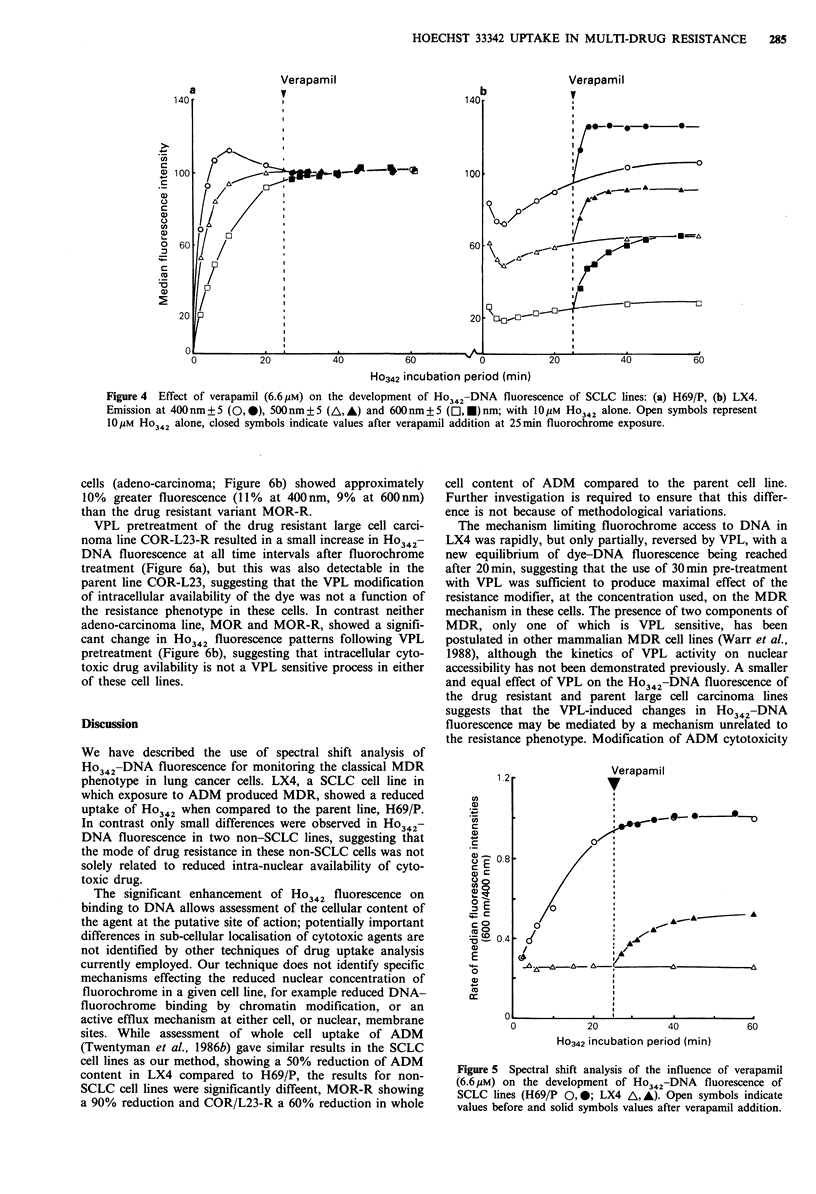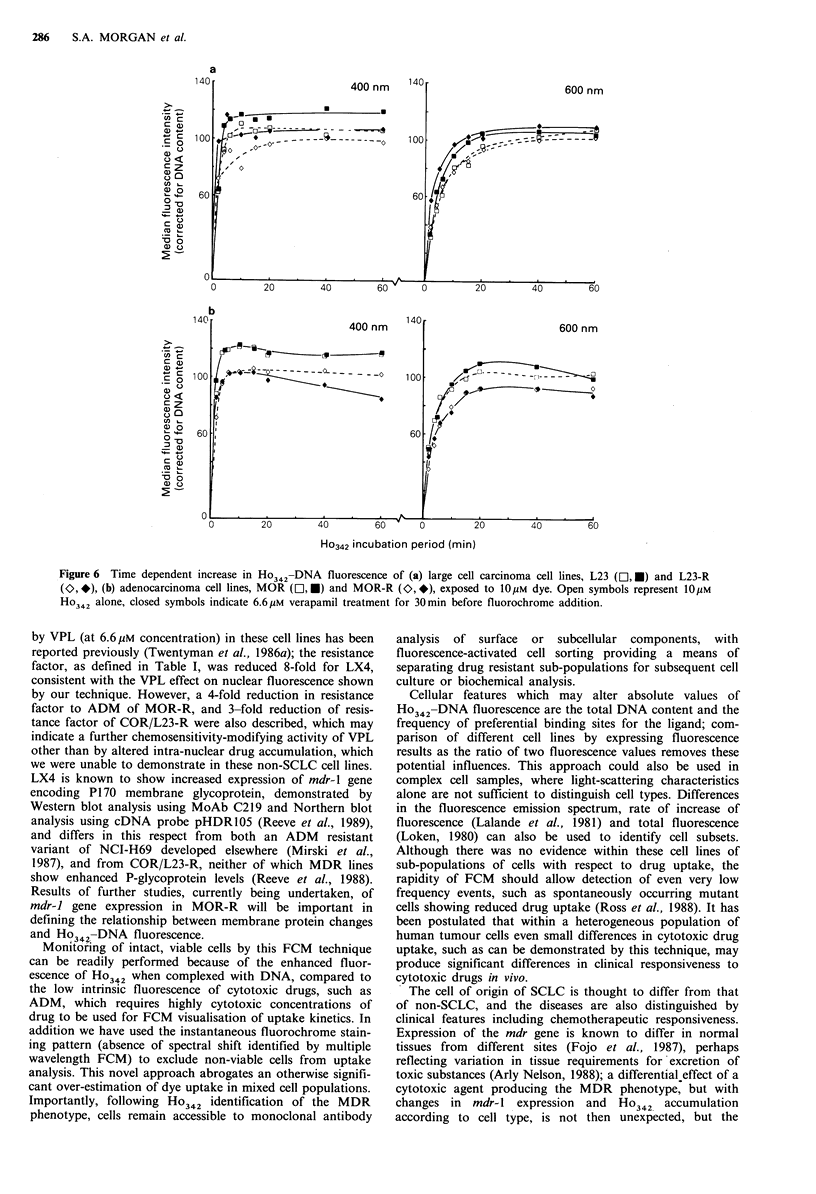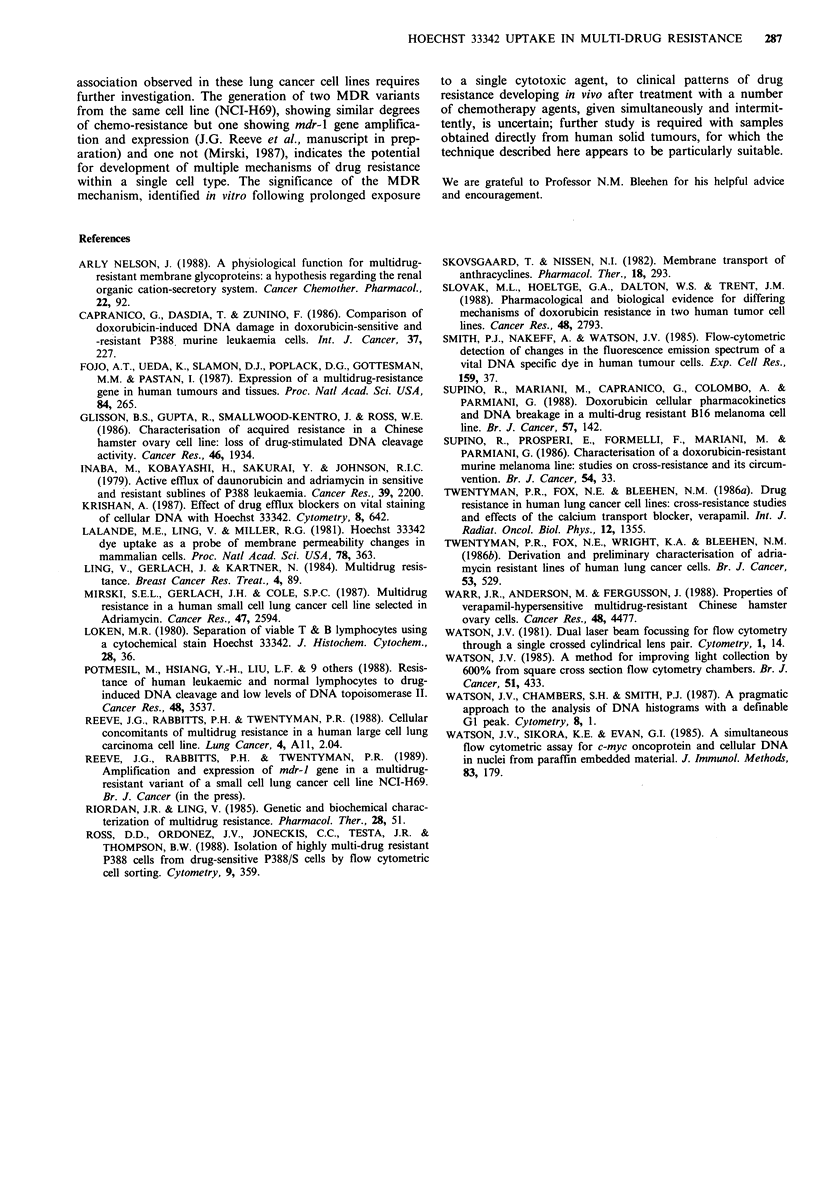# Flow cytometric analysis of Hoechst 33342 uptake as an indicator of multi-drug resistance in human lung cancer.

**DOI:** 10.1038/bjc.1989.271

**Published:** 1989-09

**Authors:** S. A. Morgan, J. V. Watson, P. R. Twentyman, P. J. Smith

**Affiliations:** Clinical Oncology and Radiotherapeutics Unit, Addenbrooke's Hospital, Cambridge, UK.

## Abstract

Cytotoxic drug resistance developing after chemotherapy is thought to be the main cause of treatment failure in several human tumours, including small cell lung cancer (SCLC). Cell lines showing drug resistance following prolonged exposure to a single agent frequently acquire resistance to several functionally unrelated drugs, the phenomenon of multi-drug resistance (MDR). Classical MDR is thought to arise from changes effecting a reduction in intracellular availability of cytotoxic drugs. We describe a flow cytometry (FCM) technique to monitor the MDR phenotype in drug resistant variants of SCLC and non-SCLC cell lines. The technique is based on a multiparametric analysis of the nuclear binding of a model chemotherapeutic agent, the fluorescent dye Hoechst 33342 (Ho342), which is capable of supra-vital staining of DNA in intact, viable cells. A laboratory derived drug resistant SCLC cell line, H69/LX4, showed a significant (30%) reduction in nuclear binding compared to the parental line H69/P. Exposure to verapamil (VPL) rapidly increased (within 2 min) nuclear binding of Ho342, and the new equilibrium of nuclear staining, attained within 20 min, remained lower than the level achieved in the parental cell line, suggesting some ability of H69/LX4 to limit the effect of the drug efflux blocker. A drug resistant large cell carcinoma line showed only a small reduction (10%) in nuclear binding when compared to the parent line, and this difference was not altered by VPL. A drug resistant adenocarcinoma line showed less than 10% difference in nuclear binding compared with the parental line and neither line was significantly affected by VPL treatment. Our findings suggest that different mechanisms of resistance may occur in lung tumours of different tissue types. This technique may be extended to the rapid and direct examination of biopsy specimens of human solid tumours for evidence of multi-drug resistance.


Br. J. Cancer (1989), 60, 282-287 © The Macmillan Press Ltd., 1989

### Flow cytometric analysis of Hoechst 33342 uptake as an indicator of multi-drug resistance in human lung cancer

S.A. Morgan, J.V. Watson, P.R. Twentyman & P.J. Smith

*Clinical Oncology and Radiotherapeutics Unit, MRC, Addenbrooke's Hospital, Hills Road, Cambridge CB2 2QQ, UK.*

**Summary** Cytotoxic drug resistance developing after chemotherapy is thought to be the main cause of treatment failure in several human tumours, including small cell lung cancer (SCLC). Cell lines showing drug resistance following prolonged exposure to a single agent frequently acquire resistance to several functionally unrelated drugs, the phenomenon of multi-drug resistance (MDR). Classical MDR is thought to arise from changes effecting a reduction in intracellular availability of cytotoxic drugs. We describe a flow cytometry (FCM) technique to monitor the MDR phenotype in drug resistant variants of SCLC and non-SCLC cell lines. The technique is based on a multiparametric analysis of the nuclear binding of a model chemotherapeutic agent, the fluorescent dye Hoechst 33342 ($Ho_{342}$), which is capable of supra-vital staining of DNA in intact, viable cells. A laboratory derived drug resistant SCLC cell line, H69/LX4, showed a significant (30%) reduction in nuclear binding compared to the parental line H69/P. Exposure to verapamil (VPL) rapidly increased (within 2 min) nuclear binding of $Ho_{342}$, and the new equilibrium of nuclear staining, attained within 20 min, remained lower than the level achieved in the parental cell line, suggesting some ability of H69/LX4 to limit the effect of the drug efflux blocker. A drug resistant large cell carcinoma line showed only a small reduction (10%) in nuclear binding when compared to the parent line, and this difference was not altered by VPL. A drug resistant adenocarcinoma line showed less than 10% difference in nuclear binding compared with the parental line and neither line was significantly affected by VPL treatment. Our findings suggest that different mechanisms of resistance may occur in lung tumours of different tissue types. This technique may be extended to the rapid and direct examination of biopsy specimens of human solid tumours for evidence of multi-drug resistance.

Emergence of resistance to initially effective cytotoxic drugs is a major cause of treatment failure in small cell lung cancer, a disease characterised by high initial response rates to chemotherapy but ultimately low incidence of long term control. Frequently post-chemotherapy relapse is refractory even to cytotoxic drugs to which the tumour has not previously been exposed. *In vitro* models of drug resistance have provided much information on the cellular pathways which may be involved. Postulated mechanisms include: reduced drug accumulation (Skovsgaard & Nissen, 1982; Inaba *et al.*, 1979), altered intracellular distribution of drug (Supino *et al.*, 1986), reduced DNA cleavage (Glisson *et al.*, 1986; Capranico *et al.*, 1986; Supino *et al.*, 1988) or altered drug activity at other target sites (Potmesil *et al.*, 1988). The development of 'classical' multi-drug resistance (classical MDR) is a frequent outcome after selection of resistance *in vitro*, such a phenotype is thought to reflect the increased efflux of cytotoxic drugs by an energy-dependent transport mechanism involving membrane located P-glycoprotein (Ling *et al.*, 1974), a finding which has been confirmed in several (Riordan & Ling, 1985), but not all (Slovak *et al.*, 1988; Mirski *et al.*, 1987), cell lines showing MDR.

The analysis of patterns of drug uptake is clearly fundamental to the study of chemo-responsiveness of tumour populations, yet whole population extraction methods cannot identify important differences in intracellular location of the drug and are inappropriate where there is possible cellular heterogeneity. This paper considers the use of multiparameter flow cytometry (FCM) to evaluate the MDR phenotype in tumour cell populations, using a technique to detect anomalies in nuclear accumulation of a model chemotherapeutic agent, Hoechst dye 33342 ($Ho_{342}$). $Ho_{342}$ is a vital, DNA-specific, *bis*-benzimidazole dye, which shows considerable fluorescence enhancement upon binding noncovalently to the minor groove of the double helix, permitting the flow cytometric determination of dye uptake and subsequent interaction with a defined intracellular target. The uptake of this agent has been shown to parallel that of a number of cytotoxic drugs involved in MDR and to correlate with cellular sensitivity to these drugs (Lalande *et al.*, 1981); it is also modified by those agents that are known to partially overcome the MDR phenotype (calmodulin inhibitors and $Ca^{2+}$ channel blockers; Krishan, 1987).

The $Ho_{342}$–DNA fluorescence emission spectrum is known to show a shift to longer wavelengths as the dye:DNA-phosphate ratio increases (Smith *et al.*, 1985), reflecting a reduction in binding energy that occurs at higher concentrations of DNA-bound dye due to co-operativity between ligand molecules. We have used the shift in fluorescence emission spectrum which occurs at early time points after $Ho_{342}$ treatment in the analysis of (i) changes in the intracellular availability of this model cytotoxic agent in human SCLC and non-SCLC lung cancer cell lines, and (ii) the responsiveness of drug resistant cells to verapamil (VPL).

### Materials and methods

### *Cell lines and culture conditions*

Stock cultures of human cell lines were maintained in RPMI medium supplemented with 10% fetal calf serum, penicillin and streptomycin (all Gibco Europe Ltd), at 37°C in an 8% $CO_2$/92% air mixture. The origins and characteristics of the cell lines are shown in Table I. NCI-H69/P (H69/P) and H69/LX4 (LX4) grew as free-floating aggregates; COR-L23, MOR and their drug resistant variants grew as adherent monolayers. The procedure for the derivation of drug resistant cell lines in this laboratory, by step-wise exposure to increasing adriamycin (ADM) concentrations, has been reported previously (Twentyman *et al.*, 1986*b*). Resistant cell lines were maintained under selective conditions with ADM (Farmitalia Carlo Erba) at the concentrations shown. Cultures were grown in non-selective medium for 24–72 h before an experiment. (Similar results were obtained for cells maintained under non-selective conditions for up to 7 days – results not shown.)

### *Preparation and treatment of cultures*

Cells were disaggregated using a 15 min treatment with 0.4% trypsin and 0.02% versene (Gibco Biocult) at 37°C. No differences were seen in subsequent $Ho_{342}$, uptake of H69/P cells as a result of this preparatory method compared to gentle disaggregation of loose aggregates by pipetting (results not shown). Cells were washed and resuspended at $2 \times 10^5$ cells $ml^{-1}$ in complete culture medium supplemented with 10 mM Hepes (*N*-2-hydroxyethylpiperazine-*N*-2-ethane sul-

Correspondence: S.A. Morgan.
Received 13 January 1989, and in revised form, 5 April 1989.

**Figure 1** Time dependent increase in $Ho_{342}$–DNA fluorescence of SCLC cells (H69/P ○; LX4 △) exposed to 10 µM dye. Fluorescence values expressed as a percentage of the value obtained for H69/P cells at 40 min as detailed in the text. Data points are arithmetic means of six experiments; error bars show ±1 s.e.m.

phonic acid), and held under standard culture conditions for a minimum of 30 min before further manipulation.

*Flow cytometric analyses*

*Cell cycle analysis* A rapid one-step DNA-staining technique using ethidium bromide (50 µg ml$^{-1}$ Triton X-100 0.125%, ribonuclease 0.5 µg ml$^{-1}$; 10 min at room temperature) was used to measure DNA content by reference to human peripheral blood leucocytes stained concurrently (mean DNA content of 8.3 pg cell$^{-1}$; Smith, 1985). Cell cycle distribution was analysed by the computer algorithm described previously (Watson *et al.*, 1987).

*Nuclear $Ho_{342}$ accumulation* The exact concentrations of filter sterilised stock solutions of $Ho_{342}$ (CP Laboratories, Bishop's Stortford, UK) were determined spectrophotometrically (molar extinction coefficient $4.1 \times 10^4 M^{-1} cm^{-1}$ at 340 nm, pH 7.0). All treatments were at 10 µM dye concentration and at 37°C. Cells were exposed to 3.3 µg ml$^{-1}$ (6.6 µM) VPL (Abbott Laboratories, Queensborough, Kent) either for 30 min before addition of $Ho_{342}$, or at 25 min after $Ho_{342}$ treatment.

Cell suspensions were analyzed directly after fluorochrome treatment using a flow cytometer, details of which have been published previously (Watson, 1981), incorporating a high light collection efficiency flow chamber (Watson, 1985) considered necessary for these experiments. Briefly, fluorescence excitation was by a krypton laser tuned to 337 nm (200 mW light power). Five parameters were recorded: 90° light scatter (<370 nm) acting as the master trigger, forward light scatter and three fluorescence channels monitoring selected regions of the $Ho_{342}$-DNA emission spectrum (400 nm, 500 nm and 600 nm, all ±5 nm). Sub-cellular debris and cell clumps were excluded by electronic gating on the basis of light-scatter signals and pulse-shape analysis (Watson *et al.*, 1985). Analysis was performed on two-dimensional displays of fluorescence data (400 nm *versus* 600 nm) for each sample by gating on regions of interest and obtaining median fluorescence values. To allow comparison of a series of $Ho_{342}$ uptake experiments a standard sample of H69/P cells (after 40 min fluorochrome treatment) was prepared for each analysis, and median fluorescence results are quoted normalised to this control value.

### Results

The DNA content and cell cycle distribution of asynchronously growing parent and resistant lines of both SCLC and adenocarcinoma types were similar (Table I). The large cell carcinoma line COR-L23 showed a greater $G_2/M$ proportion and COR-L23R a greater $G_1$ DNA content than H69/P, so that the mean cellular DNA content of these lines was, respectively, 1.5 and 1.8 times that of H69/P. As the level of $Ho_{342}$ fluorescence is dependent on cellular DNA content – as well as the intranuclear fluorochrome content – the fluorescence values shown are corrected for the mean cellular DNA content of the whole cell population with respect to H69/P.

The increase in nuclear fluorescence of H69/P and LX4 cells as a function of exposure period to $Ho_{342}$ is shown in Figure 1. The drug resistant cell line showed a slower

**Table I** Tumour cell line characteristics

| *Cell line designation* | *Origin* | *ADM resistance*[a] | *Cell cycle distribution*[b] | | | *Mean DNA content*[b] (*pg DNA cell*$^{-1}$) | |
|---|---|---|---|---|---|---|---|
| | | | *%G$_1$* | *%S* | *%G$_2$/M* | *G$_1$* | *Whole population* |
| NCI/H69-P | SCLC[c] | – | 56 | 31 | 13 | 12.0 | 12.8 |
| NCI/H69-LX4 | Resistant SCLC[c] | + (85) | 45 | 40 | 15 | 11.1 | 12.3 |
| MOR | AdenoCA[d] | – | 57 | 32 | 11 | 11.1 | 12.9 |
| MOR-R | Resistant AdenoCA[d] | + (12) | 44 | 33 | 23 | 10.8 | 11.4 |
| COR-L23 | Large cell CA[d] | – | 22 | 34 | 44 | 11.1 | 19.8 |
| COR-L23-R | Resistant large cell CA[d] | + (11) | 39 | 41 | 20 | 16.7 | 23.4 |

[a]Figures in brackets indicate resistance factor to Adriamycin (Twentyman *et al.*, 1986*b*) determined as the ratio of Adriamycin doses, for resistant compared to parent cell line, required to suppress cell numbers to 20% of control values with continuous drug exposure. [b]Determined by FCM, with reference to human leucocyte standard. [c]SCLC: small cell lung cancer, cultured as free-floating aggregates. [d]CA: carcinoma, cultured as attached monolayers.

**Figure 2** Spectral shift of $Ho_{342}$-DNA fluorescence with time after dye exposure for SCLC cells. Results represent means of ratios derived from six experiments (standardised to the H69/P control; see legend to Figure 1). Symbols as in Figure 1.

development of fluorescence at all wavelengths (only 400 nm and 600 nm are shown) which was most pronounced at longer wavelengths (50% reduction at 600 nm compared with 10% at 400 nm). By considering the ratio of two fluorescence values the influence of cellular DNA content on total fluorescence is removed and different cell lines can be directly compared on the basis of the rate at which ligand is binding to DNA (i.e. the spectral shift). This change in fluorescence spectrum, due to altered cellular content of the dye, is demonstrated by considering the ratio of the 600 nm value to that at 400 nm (Figure 2). After $Ho_{342}$ treatment of H69/P, a spectral shift occurred during the initial 20 min time interval, due to increasing dye–DNA binding, whereas the drug resistant line LX4 showed no alteration of the fluorescence ratio during this period, reflecting a significant limitation on the intracellular availability of the ligand for interaction with DNA.

It was noted that a small proportion of cells in all preparations showed instantaneous (< 1 min) development of fluorescence at both wavelengths simultaneously. These cells are considered to be non-viable because they are unable to provide any barrier to $Ho_{342}$-DNA binding and simultaneously show efficient staining with propidium iodide (data not shown). The effect of even small numbers of these non-viable cells was both to increase the whole population fluorescence and to increase the apparent ratio of the 600 nm/400 nm fluorescence emission, particularly at early time intervals after fluorochrome treatment. This significantly alters the fluorescence ratio of the drug resistant line LX4 because of the relatively low levels of absolute fluorescence, and would result in an overestimate of drug uptake (e.g. the 2 min time points in Figure 1). In all subsequent analyses presented here cells showing instantaneous spectral shift patterns after $Ho_{342}$ treatment have been excluded by dual parameter analysis as indicated in the Materials and methods.

A 30 min pretreatment of LX4 cells with VPL resulted in increased $Ho_{342}$–DNA fluorescence at all wavelengths on subsequent fluorochrome treatment (see Figure 3), from 30 to 50% that of the H69/P control, indicating increased intracellular availability of the fluorochrome; no significant change was observed with H69/P given this treatment. The time course of the effect of VPL on LX4 was clearly demonstrated by adding VPL after 25 min prior exposure to fluorochrome, at which time both sensitive and ADM-resistant lines had reached an equilibrium for $Ho_{342}$-DNA fluorescence (Figure 4). Under these conditions the VPL-induced increase in fluorescence of LX4 occurred at a rate as rapid as the initial phase of $Ho_{342}$ uptake by H69/P. The observed increase in the fluorescence ratio of LX4 reached a new equilibrium value below that of the parent cell line within 20 minutes of $Ho_{342}$ addition, after which there was no further change despite continued exposure to both VPL and dye (Figure 5); clearly the treatment did not fully reverse the phenotype of reduced intracellular availability of $Ho_{342}$. Further experiments demonstrated the fluorescence shift to be VPL concentration dependent, but at higher concentrations abnormalities in the forward scatter characteristic, and reduction in cell number that also occurred, suggested that VPL was having toxic effects (results not shown).

We have attempted to demonstrate a similar mechanism of reduced $Ho_{342}$–DNA binding in drug resistant non-SCLC lines COR-L23-R and MOR-R compared to the parent lines, from which they had been derived by intermittent ADM exposure in an identical manner to LX4. Both were found to show much smaller differences in $Ho_{342}$–DNA fluorescence between the parent and corresponding drug resistant lines. COR/L23 cells (large cell carcinoma; Figure 6a) showed a plateau fluorescence value which was only slightly greater than COR/L23-R (7% at 400 nm and 2% at 600 nm); MOR

**Figure 3** Effect of verapamil pre-treatment on $Ho_{342}$–DNA fluorescence of SCLC cells (○, ● H69/P; △, ▲ LX4); median fluorescence values (standardised to the H69/P control). Open symbols represent 10 μM $Ho_{342}$ alone, closed symbols indicate 6.6 μM verapamil for 30 min before fluorochrome addition.

**Figure 4** Effect of verapamil (6.6 $\mu$M) on the development of $Ho_{342}$–DNA fluorescence of SCLC lines: (**a**) H69/P, (**b**) LX4. Emission at 400 nm ± 5 (○, ●), 500 nm ± 5 (△, ▲) and 600 nm ± 5 (□, ■) nm; with 10 $\mu$M $Ho_{342}$ alone. Open symbols represent 10 $\mu$M $Ho_{342}$ alone, closed symbols indicate values after verapamil addition at 25 min fluorochrome exposure.

cells (adeno-carcinoma; Figure 6b) showed approximately 10% greater fluorescence (11% at 400 nm, 9% at 600 nm) than the drug resistant variant MOR-R.

VPL pretreatment of the drug resistant large cell carcinoma line COR-L23-R resulted in a small increase in $Ho_{342}$–DNA fluorescence at all time intervals after fluorochrome treatment (Figure 6a), but this was also detectable in the parent line COR-L23, suggesting that the VPL modification of intracellular availability of the dye was not a function of the resistance phenotype in these cells. In contrast neither adeno-carcinoma line, MOR and MOR-R, showed a significant change in $Ho_{342}$ fluorescence patterns following VPL pretreatment (Figure 6b), suggesting that intracellular cytotoxic drug avilability is not a VPL sensitive process in either of these cell lines.

### Discussion

We have described the use of spectral shift analysis of $Ho_{342}$–DNA fluorescence for monitoring the classical MDR phenotype in lung cancer cells. LX4, a SCLC cell line in which exposure to ADM produced MDR, showed a reduced uptake of $Ho_{342}$ when compared to the parent line, H69/P. In contrast only small differences were observed in $Ho_{342}$–DNA fluorescence in two non–SCLC lines, suggesting that the mode of drug resistance in these non-SCLC cells was not solely related to reduced intra-nuclear availability of cytotoxic drug.

The significant enhancement of $Ho_{342}$ fluorescence on binding to DNA allows assessment of the cellular content of the agent at the putative site of action; potentially important differences in sub-cellular localisation of cytotoxic agents are not identified by other techniques of drug uptake analysis currently employed. Our technique does not identify specific mechanisms effecting the reduced nuclear concentration of fluorochrome in a given cell line, for example reduced DNA–fluorochrome binding by chromatin modification, or an active efflux mechanism at either cell, or nuclear, membrane sites. While assessment of whole cell uptake of ADM (Twentyman *et al.*, 1986*b*) gave similar results in the SCLC cell lines as our method, showing a 50% reduction of ADM content in LX4 compared to H69/P, the results for non-SCLC cell lines were significantly diffeent, MOR-R showing a 90% reduction and COR/L23-R a 60% reduction in whole cell content of ADM compared to the parent cell line. Further investigation is required to ensure that this difference is not because of methodological variations.

The mechanism limiting fluorochrome access to DNA in LX4 was rapidly, but only partially, reversed by VPL, with a new equilibrium of dye–DNA fluorescence being reached after 20 min, suggesting that the use of 30 min pre-treatment with VPL was sufficient to produce maximal effect of the resistance modifier, at the concentration used, on the MDR mechanism in these cells. The presence of two components of MDR, only one of which is VPL sensitive, has been postulated in other mammalian MDR cell lines (Warr *et al.*, 1988), although the kinetics of VPL activity on nuclear accessibility has not been demonstrated previously. A smaller and equal effect of VPL on the $Ho_{342}$–DNA fluorescence of the drug resistant and parent large cell carcinoma lines suggests that the VPL-induced changes in $Ho_{342}$–DNA fluorescence may be mediated by a mechanism unrelated to the resistance phenotype. Modification of ADM cytotoxicity

**Figure 5** Spectral shift analysis of the influence of verapamil (6.6 $\mu$M) on the development of $Ho_{342}$–DNA fluorescence of SCLC lines (H69/P ○, ●; LX4 △, ▲). Open symbols indicate values before and solid symbols values after verapamil addition.

**Figure 6** Time dependent increase in $Ho_{342}$–DNA fluorescence of (**a**) large cell carcinoma cell lines, L23 (□, ■) and L23-R (◇, ◆), (**b**) adenocarcinoma cell lines, MOR (□, ■) and MOR-R (◇, ◆), exposed to 10 μM dye. Open symbols represent 10 μM $Ho_{342}$ alone, closed symbols indicate 6.6 μM verapamil treatment for 30 min before fluorochrome addition.

by VPL (at 6.6 μM concentration) in these cell lines has been reported previously (Twentyman *et al.*, 1986*a*); the resistance factor, as defined in Table I, was reduced 8-fold for LX4, consistent with the VPL effect on nuclear fluorescence shown by our technique. However, a 4-fold reduction in resistance factor to ADM of MOR-R, and 3–fold reduction of resistance factor of COR/L23-R were also described, which may indicate a further chemosensitivity-modifying activity of VPL other than by altered intra-nuclear drug accumulation, which we were unable to demonstrate in these non-SCLC cell lines. LX4 is known to show increased expression of *mdr*-1 gene encoding P170 membrane glycoprotein, demonstrated by Western blot analysis using MoAb C219 and Northern blot analysis using cDNA probe pHDR105 (Reeve *et al.*, 1989), and differs in this respect from both an ADM resistant variant of NCI-H69 developed elsewhere (Mirski *et al.*, 1987), and from COR/L23-R, neither of which MDR lines show enhanced P-glycoprotein levels (Reeve *et al.*, 1988). Results of further studies, currently being undertaken, of *mdr-1* gene expression in MOR-R will be important in defining the relationship between membrane protein changes and $Ho_{342}$–DNA fluorescence.

Monitoring of intact, viable cells by this FCM technique can be readily performed because of the enhanced fluorescence of $Ho_{342}$ when complexed with DNA, compared to the low intrinsic fluorescence of cytotoxic drugs, such as ADM, which requires highly cytotoxic concentrations of drug to be used for FCM visualisation of uptake kinetics. In addition we have used the instantaneous fluorochrome staining pattern (absence of spectral shift identified by multiple wavelength FCM) to exclude non-viable cells from uptake analysis. This novel approach abrogates an otherwise significant over-estimation of dye uptake in mixed cell populations. Importantly, following $Ho_{342}$ identification of the MDR phenotype, cells remain accessible to monoclonal antibody analysis of surface or subcellular components, with fluorescence-activated cell sorting providing a means of separating drug resistant sub-populations for subsequent cell culture or biochemical analysis.

Cellular features which may alter absolute values of $Ho_{342}$–DNA fluorescence are the total DNA content and the frequency of preferential binding sites for the ligand; comparison of different cell lines by expressing fluorescence results as the ratio of two fluorescence values removes these potential influences. This approach could also be used in complex cell samples, where light-scattering characteristics alone are not sufficient to distinguish cell types. Differences in the fluorescence emission spectrum, rate of increase of fluorescence (Lalande *et al.*, 1981) and total fluorescence (Loken, 1980) can also be used to identify cell subsets. Although there was no evidence within these cell lines of sub-populations of cells with respect to drug uptake, the rapidity of FCM should allow detection of even very low frequency events, such as spontaneously occurring mutant cells showing reduced drug uptake (Ross *et al.*, 1988). It has been postulated that within a heterogeneous population of human tumour cells even small differences in cytotoxic drug uptake, such as can be demonstrated by this technique, may produce significant differences in clinical responsiveness to cytotoxic drugs *in vivo*.

The cell of origin of SCLC is thought to differ from that of non-SCLC, and the diseases are also distinguished by clinical features including chemotherapeutic responsiveness. Expression of the *mdr* gene is known to differ in normal tissues from different sites (Fojo *et al.*, 1987), perhaps reflecting variation in tissue requirements for excretion of toxic substances (Arly Nelson, 1988); a differential effect of a cytotoxic agent producing the MDR phenotype, but with changes in *mdr*-1 expression and $Ho_{342}$ accumulation according to cell type, is not then unexpected, but the association observed in these lung cancer cell lines requires further investigation. The generation of two MDR variants from the same cell line (NCI-H69), showing similar degrees of chemo-resistance but one showing *mdr*-1 gene amplification and expression (J.G. Reeve *et al.*, manuscript in preparation) and one not (Mirski, 1987), indicates the potential for development of multiple mechanisms of drug resistance within a single cell type. The significance of the MDR mechanism, identified *in vitro* following prolonged exposure to a single cytotoxic agent, to clinical patterns of drug resistance developing *in vivo* after treatment with a number of chemotherapy agents, given simultaneously and intermittently, is uncertain; further study is required with samples obtained directly from human solid tumours, for which the technique described here appears to be particularly suitable.

We are grateful to Professor N.M. Bleehen for his helpful advice and encouragement.

**References**

ARLY NELSON, J. (1988). A physiological function for multidrug-resistant membrane glycoproteins: a hypothesis regarding the renal organic cation-secretory system. *Cancer Chemother. Pharmacol.*, **22,** 92.

CAPRANICO, G., DASDIA, T. & ZUNINO, F. (1986). Comparison of doxorubicin-induced DNA damage in doxorubicin-sensitive and -resistant P388 murine leukaemia cells. *Int. J. Cancer,* **37,** 227.

FOJO, A.T., UEDA, K., SLAMON, D.J., POPLACK, D.G., GOTTESMAN, M.M. & PASTAN, I. (1987). Expression of a multidrug-resistance gene in human tumours and tissues. *Proc. Natl Acad. Sci. USA,* **84,** 265.

GLISSON, B.S., GUPTA, R., SMALLWOOD-KENTRO, J. & ROSS, W.E. (1986). Characterisation of acquired resistance in a Chinese hamster ovary cell line: loss of drug-stimulated DNA cleavage activity. *Cancer Res.*, **46,** 1934.

INABA, M., KOBAYASHI, H., SAKURAI, Y. & JOHNSON, R.I.C. (1979). Active efflux of daunorubicin and adriamycin in sensitive and resistant sublines of P388 leukaemia. *Cancer Res.*, **39,** 2200.

KRISHAN, A. (1987). Effect of drug efflux blockers on vital staining of cellular DNA with Hoechst 33342. *Cytometry,* **8,** 642.

LALANDE, M.E., LING, V. & MILLER, R.G. (1981). Hoechst 33342 dye uptake as a probe of membrane permeability changes in mammalian cells. *Proc. Natl Acad. Sci. USA,* **78,** 363.

LING, V., GERLACH, J. & KARTNER, N. (1984). Multidrug resistance. *Breast Cancer Res. Treat.*, **4,** 89.

MIRSKI, S.E.L., GERLACH, J.H. & COLE, S.P.C. (1987). Multidrug resistance in a human small cell lung cancer cell line selected in Adriamycin. *Cancer Res.*, **47,** 2594.

LOKEN, M.R. (1980). Separation of viable T & B lymphocytes using a cytochemical stain Hoechst 33342. *J. Histochem. Cytochem.*, **28,** 36.

POTMESIL, M., HSIANG, Y.-H., LIU, L.F. & 9 others (1988). Resistance of human leukaemic and normal lymphocytes to drug-induced DNA cleavage and low levels of DNA topoisomerase II. *Cancer Res.*, **48,** 3537.

REEVE, J.G., RABBITTS, P.H. & TWENTYMAN, P.R. (1988). Cellular concomitants of multidrug resistance in a human large cell lung carcinoma cell line. *Lung Cancer,* **4,** A11, 2.04.

REEVE, J.G., RABBITTS, P.H. & TWENTYMAN, P.R. (1989). Amplification and expression of *mdr-1* gene in a multidrug-resistant variant of a small cell lung cancer cell line NCI-H69. *Br. J. Cancer* (in the press).

RIORDAN, J.R. & LING, V. (1985). Genetic and biochemical characterization of multidrug resistance. *Pharmacol. Ther.*, **28,** 51.

ROSS, D.D., ORDONEZ, J.V., JONECKIS, C.C., TESTA, J.R. & THOMPSON, B.W. (1988). Isolation of highly multi-drug resistant P388 cells from drug-sensitive P388/S cells by flow cytometric cell sorting. *Cytometry,* **9,** 359.

SKOVSGAARD, T. & NISSEN, N.I. (1982). Membrane transport of anthracyclines. *Pharmacol. Ther.*, **18,** 293.

SLOVAK, M.L., HOELTGE, G.A., DALTON, W.S. & TRENT, J.M. (1988). Pharmacological and biological evidence for differing mechanisms of doxorubicin resistance in two human tumor cell lines. *Cancer Res.*, **48,** 2793.

SMITH, P.J., NAKEFF, A. & WATSON, J.V. (1985). Flow-cytometric detection of changes in the fluorescence emission spectrum of a vital DNA specific dye in human tumour cells. *Exp. Cell Res.*, **159,** 37.

SUPINO, R., MARIANI, M., CAPRANICO, G., COLOMBO, A. & PARMIANI, G. (1988). Doxorubicin cellular pharmacokinetics and DNA breakage in a multi-drug resistant B16 melanoma cell line. *Br. J. Cancer,* **57,** 142.

SUPINO, R., PROSPERI, E., FORMELLI, F., MARIANI, M. & PARMIANI, G. (1986). Characterisation of a doxorubicin-resistant murine melanoma line: studies on cross-resistance and its circumvention. *Br. J. Cancer,* **54,** 33.

TWENTYMAN, P.R., FOX, N.E. & BLEEHEN, N.M. (1986*a*). Drug resistance in human lung cancer cell lines: cross-resistance studies and effects of the calcium transport blocker, verapamil. *Int. J. Radiat. Oncol. Biol. Phys.*, **12,** 1355.

TWENTYMAN, P.R., FOX, N.E., WRIGHT, K.A. & BLEEHEN, N.M. (1986*b*). Derivation and preliminary characterisation of adriamycin resistant lines of human lung cancer cells. *Br. J. Cancer,* **53,** 529.

WARR, J.R., ANDERSON, M. & FERGUSSON, J. (1988). Properties of verapamil-hypersensitive multidrug-resistant Chinese hamster ovary cells. *Cancer Res.*, **48,** 4477.

WATSON, J.V. (1981). Dual laser beam focussing for flow cytometry through a single crossed cylindrical lens pair. *Cytometry,* **1,** 14.

WATSON, J.V. (1985). A method for improving light collection by 600% from square cross section flow cytometry chambers. *Br. J. Cancer,* **51,** 433.

WATSON, J.V., CHAMBERS, S.H. & SMITH, P.J. (1987). A pragmatic approach to the analysis of DNA histograms with a definable G1 peak. *Cytometry,* **8,** 1.

WATSON, J.V., SIKORA, K.E. & EVAN, G.I. (1985). A simultaneous flow cytometric assay for *c-myc* oncoprotein and cellular DNA in nuclei from paraffin embedded material. *J. Immunol. Methods,* **83,** 179.